# Examining changes in school vending machine beverage availability and sugar-sweetened beverage intake among Canadian adolescents participating in the COMPASS study: a longitudinal assessment of provincial school nutrition policy compliance and effectiveness

**DOI:** 10.1186/s12966-018-0754-5

**Published:** 2018-11-27

**Authors:** Katelyn M. Godin, David Hammond, Ashok Chaurasia, Scott T. Leatherdale

**Affiliations:** School of Public Health and Health Systems, University of Waterloo, 200 University Avenue West, Waterloo, ON N2L 3G1 Canada

**Keywords:** School nutrition policy, Sugar-sweetened beverages, Canada, Adolescents, Schools, Vending machines, Longitudinal study, Dietary assessment

## Abstract

**Background:**

School nutrition policies can encourage restrictions in sugar-sweetened beverage (SSB) availability in school food outlets in order to discourage students’ SSB intake. The main objective was to examine how beverage availability in school vending machines changes over three school years across schools in distinct school nutrition policy contexts. Secondary objectives were to examine how students’ weekday SSB intake varies with time and identify longitudinal associations between beverage availability and SSB intake.

**Methods:**

This longitudinal study used data from the COMPASS study (2013/14–2015/16), representing 7679 students from 78 Canadian secondary schools and three provincial school nutrition policy contexts (Alberta – voluntary guidelines, Ontario public – mandatory guidelines, and Ontario private schools – no guidelines). We assessed availability of 10 beverage categories in schools’ vending machines via the COMPASS School Environment Application and participants’ intake of three SSB varieties (soft drinks, sweetened coffees/teas, and energy drinks) via a questionnaire. Hierarchical regression models were used to examine whether: i) progression of time and policy group were associated with beverage availability; and, ii) beverage availability was associated with students’ SSB intake.

**Results:**

Ontario public schools were significantly less likely than the other policy groups to serve SSBs in their vending machines, with the exception of flavoured milks. Vending machine beverage availability was consistent over time. Participants’ overall SSB intake remained relatively stable; reductions in soft drink intake were partially offset by increased sweetened coffee/tea consumption. Relative to Ontario public schools, attending school in Alberta was associated with more frequent energy drink intake and overall SSB intake whereas attending an Ontario private school was associated with less frequent soft drink intake, with no differences in overall SSB intake. Few beverage availability variables were significantly associated with participants’ SSB intake.

**Conclusions:**

Mandatory provincial school nutrition policies were predictive of more limited SSB availability in school vending machines. SSB intake was significantly lower in Ontario public and private schools, although we did not detect a direct association between SSB consumption and availability. The findings provide support for mandatory school nutrition policies, as well as the need for comprehensive school- and broader population-level efforts to reduce SSB intake.

**Electronic supplementary material:**

The online version of this article (10.1186/s12966-018-0754-5) contains supplementary material, which is available to authorized users.

## Background

Adolescents are the largest consumers of sugar-sweetened beverages (SSBs) in Canada [1], mirroring trends in other countries like Great Britain and the United States [2, 3], and many Canadian youth report daily SSB consumption [4–6]. SSBs are a category of beverages that contain added sugars, including ‘regular’ soft drinks, fruit drinks, sports drinks, energy drinks, and sweetened coffees/teas. Frequent SSB consumption is associated with an increased risk of overweight/obesity [7–9], lower intake of vitamins and nutrients [10, 11], type-2 diabetes [12, 13], and cardiovascular disease [14, 15]. SSB consumption remains high as youth progress through adolescence and increases among some subgroups (e.g., males) [14, 16], which is concerning since youths’ dietary habits often persist into adulthood [17]. As such, adolescents represent an important target for prevention efforts to reduce SSB consumption.

Schools represent a feasible and practical setting for initiatives to improve youths’ dietary behaviours, given their population coverage, the amount of time youth spend in school, and the fact that students generally eat at least one meal and/or snack during school hours [18]. Further, school-level differences account for a small, though significant proportion of the variation in adolescents’ SSB consumption [19, 20], suggesting that school characteristics may influence students’ SSB intake.

School nutrition policies indicate what foods/beverages are appropriate in schools and have been identified in the international literature as a means of means of reducing youths’ SSB intake [21, 22]. Provincial school nutrition policies exist across Canada [23] and are distinct in their scope and beverage-related guidelines. For instance, the 2012 *Alberta Nutrition Guidelines for Children & Youth (ANGCY)* offers voluntary recommendations for schools, including to avoid selling sugar-sweetened and artificially-sweetened beverages [24]. Ontario’s 2011 *Policy/program Memorandum no. 150 (P/PM 150)* includes mandatory guidelines prohibiting the sale of many SSBs in publicly-funded secondary schools, such as < 100% juice drinks, sports drinks, and “other” beverages (e.g., soft drinks) containing > 40 cal or caffeine [25]. Since Ontario private schools are not provincial government-regulated, they are not obliged to comply with *P/PM 150*.

Despite the provincial school nutrition policies’ consistent recommendation to restrict sale of SSBs, research has shown that SSBs are often still available in Canadian schools’ food outlets [26–29]. Vine et al. identified that most public secondary schools in Alberta and Ontario were non-compliant with their respective provincial school nutrition policies’ food- and beverage-related guidelines, and that compliance decreased with time due to a lack of enforcement [26]. Other studies identified a significantly greater availability of many SSBs in school vending machines in Alberta compared to Ontario [30] and more frequent SSB consumption among Albertan students [20], and suggested that these differences reflect, in part, the mandatory vs. voluntary nature of the provincial school nutrition policies in these jurisdictions.

It is plausible that greater in-school SSBs availability encourages students to consume these products, particularly given the positive association between students’ purchases from school food outlets and their SSB intake [27]. However, there is mixed evidence linking school characteristics to students’ SSB consumption; some research identifies that in-school SSB availability predicts students’ SSB intake [27], while other studies suggest that the association between these factors is limited [31–33].

There are several research gaps that warrant investigation. First, few studies have assessed beverage availability within school food outlets longitudinally [34]. Though Vine et al. examined school vending machine food and drink contents over time [26], they reported their findings as binary measures of policy compliance (i.e., compliant vs. non-compliant), which was defined differently across provinces. Given the wide range of SSBs on the market, measuring compliance as a binary outcome provides a limited account of the availability of particular SSB types, which vary in their nutritional quality. Examining the in-school availability of specific beverages types would enable a more specific and direct comparison (i.e., since the comparison is between like and like, versus compliance with distinct policies) between jurisdictions. Second, few studies have examined how beverage availability in school food outlets influences students’ SSB intake [34]. Third, despite important distinctions in provincial school nutrition policies, there have been relatively few cross-provincial examinations of school food environment characteristics and their impact on students’ dietary outcomes, particularly within the private school system [35], which fall outside of the scope of provincial school nutrition policies in Canada.

The primary objective of this study was to examine how beverage availability in school vending machines will changes over time across three groups of secondary schools that represent distinct policy contexts (Alberta, Ontario public, and Ontario private schools). Secondary objectives were to examine how students’ weekday intake of three types of SSBs (soft drinks, sweetened coffees/teas, and energy drinks) vary over time across these policy groups, and identify longitudinal associations between SSB intake and vending machine beverage availability. The general hypothesis was that, with time, students’ SSB intake and SSB availability within school vending machines increase, reflecting decreased compliance with provincial school nutrition policies.

## Methods

COMPASS (Cannabis use, Obesity Mental health Physical activity Alcohol use Smoking Sedentary behaviour) is an ongoing (2012–2021) longitudinal cohort study designed to collect hierarchical data annually from a sample of adolescents attending Canadian secondary schools. This study used data from Year 2 (2013/14), Year 3 (2014/15), and Year 4 (2015/16) of COMPASS, which are termed Waves 1–3 herein. Given the conception of COMPASS in 2012/13 and introduction of *P/PM 150* and *ANCGY* in 2011 and 2012, the present study period represents 2–5 years post-school nutrition policy implementation.

### Sample

Both school- and student-level eligibility criteria were used to generate the sample for this study. Between Waves 1–3, 91 schools participated in at least one year of COMPASS. Each school was assigned a unique identifier, which were used to link the school samples across waves. The final school sample for this study included 78 schools that had complete data on all school-level measures in Waves 1–3. These schools represented three policy groups: Alberta – *ANGCY* (*n* = 9), Ontario public schools – *P/PM 150* (*n* = 64), and Ontario private schools – control (*n* = 5). All Albertan schools were public; however, the *ANGCY* does not distinguish between publicly- vs. privately-funded schools.

Within these 78 schools, there were 8894 students that participated in COMPASS for all three waves. As described elsewhere [36], unique self-generated identification codes were used to link student-level data sets across the three waves. Reasons for non-linkage included students graduating or being newly admitted to school within Waves 1–3, students transferring schools, having a spare/free period or being otherwise absent during data collections, dropping out of school, or inaccurate data provided on the data linkage measures. Participants missing data on outcome and/or control variables (i.e., SSB intake and socio-demographic characteristics) in any of the waves were excluded from analyses (*n* = 1215, 13.7%), except those missing body mass index (BMI) data. The final sample comprised 7679 student participants from Alberta (*n* = 497), Ontario public schools (*n* = 6674), and Ontario private schools (*n* = 508).

### Data sources

All student-level data were collected through a paper-based questionnaire comprising questions on many health, social, and academic outcomes. The questionnaire previously underwent, and performed well in, validity and reliability testing [37, 38]. All students present during the data collection were able to complete the questionnaire during class, enabling collection of whole-school samples. Student participation rates ranged over the three-year period from 78.9–80.0%.

School-level data were collected through the COMPASS School Environment Application (Co-SEA) and the Desktop Mapping Technologies Inc. (DMTI) built environment resource. The Co-SEA is a mobile application containing a series of questions adapted from two previously validated audit tools designed to efficiently measure schools’ food and physical activity environments [33, 39]. The application also allows data collectors to store photos of built environment features, and include notes within the application, representing supplementary sources of direct observation data. The Co-SEA was tested in a convenience sample of schools and refined prior to being used in the COMPASS study. The DMTI provides data on the type, location, and number of various points of interest within various circular buffers surrounding schools. Further detail on these data sources is available elsewhere [40, 41].

### SSB consumption measures

The method used to derive the outcome variables was consistent with that of previous COMPASS studies of adolescents’ SSB consumption [20, 30, 42]. Participants were asked to indicate the number of days during a usual school week (0–5 days) that they consume each of the following SSB categories: (i) “sugar-sweetened beverages (soda pop, Kool-Aid, Gatorade, etc.)”; (ii) “high-energy drinks (Red Bull, Monster, Rock Star, etc.)”; and, (iii) “coffee or tea with sugar (cappuccino, Frappuccino, iced-tea, iced-coffees, etc.)”. This first SSB category (i.e., containing soda, fruit drinks, and sports drinks) is referred to as “soft drinks” herein. Participants were advised not to include diet drinks when reporting their soft drink intake.

We used participants’ responses to these questions to derive our four study outcomes related to rate of SSB consumption: (i) number of weekdays participants reported consuming each of soft drinks, (ii) sweetened coffee/teas, and (iii) energy drinks, in addition to a composite weekday SSB score. The first three outcome variables reflect distinct beverage categories, with possible values ranging from 0 to 5 days. For the fourth outcome variable, we assessed participants’ intake of all three SSB categories captured on the questionnaire through a composite SSB score. We calculated this composite score by summing participants’ weekday consumption (in days) of each category. Possible values for this score ranged from 0 (indicating no consumption of any beverage category on any day) to 15 (indicating use of all three SSB categories every weekday). This score was intended to reflect a more comprehensive measure of participants’ total SSB consumption, in addition to their consumption of discrete SSB categories.

### Vending machine beverage availability measures

We used the Co-SEA data to assess the availability of ten beverage categories (e.g., sugar-containing carbonated soft drinks, 100% juices, water, plain white milk, etc.) within schools’ vending machines. For each vending machine, data collectors counted the number of distinct (i.e., in size, flavour, cost, etc.) products within each category, irrespective of the number of slots these products occupied. For example, a vending machine containing several slots of small and large cartons of each of chocolate and strawberry milk, was counted as having four types of flavoured milk (i.e., two flavours*two sizes). Likewise, a vending machine containing cans and bottles of regular (i.e., non-diet) Pepsi, Coca-Cola, and Sprite would be counted as having six sugar-containing carbonated soft drinks (i.e., three flavours*two sizes) available. For schools with numerous beverage vending machines, we summed the number of products within each beverage category across the machines.

### Control variables

We included both student- and school-level control variables in our analyses. Student-level control variables were gender, grade, ethnicity, weight status [i.e., BMI (kg/m2) category based on self-reported height and weight, and World Health Organizations classifications, adjusted for age and sex [43]], personal weekly spending money, truancy, and weight goal. We defined these variables in a manner that is consistent with previous COMPASS studies [20, 30].

School-level control variables were policy group, geographic location, school neighbourhood median household income, presence of a school cafeteria, presence of a school tuck shop (i.e., store that typically has snacks available for sale), and presence of three types of food outlets within the school neighbourhood (restaurants, variety stores, food/grocery stores). Geographic location categories were consistent with Statistics Canada’s definitions along the urban-rural continuum [44]. School neighbourhood median household income was derived from the 2011 National Health Survey [45], and corresponded to schools’ postal code.

We accounted for the presence of school cafeterias and tuck shops and various food outlets in the school neighbourhood as control variables, since these outlets represent other locations where students can potentially purchase SSBs. We considered access to these outlets as binary (i.e., ‘not present’ vs. ‘≥1 present). We used the Co-SEA data to assess the presence of school cafeterias and tuck shops, and the DMTI data to examine the presence of restaurants, variety stores, and food stores (i.e., grocery stores and miscellaneous food stores) within a 1-km circular buffer of schools. This buffer represents a distance that individuals can walk in 10–15 min. (e.g., during travel to/from school or during breaks) [46–48].

### Analyses

We conducted various descriptive statistics to characterize the student and school samples, including assessments of changes in vending machine beverage availability and students’ SSB-related measures across waves, stratified by policy group. We used Chi square analyses, Kruskal-Wallis tests, and Fisher’s Exact Tests to examine differences across policy groups at Wave 1 (2013/14) across categorical, non-normally distributed continuous variables, and categorical variables with small cell counts, respectively.

We used Generalized Estimating Equations (GEE) to develop hierarchical regression models to assess predictors of schools’ vending machine beverage availability (ten models; one per each beverage category) and participants’ SSB consumption (four models; one per each of the SSB consumption measures).

The models of vending machine beverage availability only contained school-level covariates, including policy group and wave (the two explanatory variables of interest), as well as geographic location, school neighbourhood median household income, and the three neighbourhood food outlet accessibility variables. We included the neighbourhood food outlet variables since previous research demonstrates that external food outlets compete with in-school food outlets [49], thus their presence may influence the product offering within the school. We modeled availability of each beverage category as a binary outcome (i.e., ‘0 drinks available’ vs. ‘≥1 drinks available’), due to concerns of small cell counts and for ease of interpretation.

The models of participants’ SSB consumption accounted for the repeated nature of COMPASS (i.e., temporal measures of the outcome variable at the student level, and spatial at the school level), and examined the association between each of the four SSB outcomes and the binary vending machine beverage availability variables. We developed a separate model for each outcome using a two-step process. First, we ran a series of univariate analyses to identify if each potential explanatory variable was independently associated with the outcome. Variables that were not statistically significantly (*P >* .2) in these univariate models at this screening stage were excluded from investigation in subsequent joint models. Second, all significant vending machine beverage availability variables from this screening stage (i.e., the vending machine beverages block of variables) were included in joint, multivariate Poisson regression models. These models included wave and all student- and school-level control variables.

We ran two additional series of models for each SSB consumption outcome variable to identify whether the addition of ‘policy group’ attenuated the effect of the vending machine beverage variables. One series of models contained the policy group effect (i.e., the policy group block) and all control variables, while the other contained both the vending machine beverages and policy group blocks, in addition to all control variables. We performed all analyses using SAS version 9.4 (SAS Institute, Cary NC).

## Results

### School characteristics and beverage availability within vending machines at baseline

Table 1 shows the school sample characteristics at Wave 1, stratified by policy group. Overall, the sample was diverse, reflecting varied geographic and socio-economic areas. There was a similar proportion of rural/small population centre schools (44.9%) and large urban population centre schools (38.4%), and most (65.5%) schools were in the $50001–75,000 neighbourhood median income range. Nearly all schools (96.2%) had at least one beverage vending machine. Most schools (85.9%) had ≥1100% fruit juice available in the vending machines, making it the most commonly available beverage, followed by water (73.1%) and diet carbonated soft drinks (71.8%). Relatively few schools had sugar-containing sports drinks (14.1%), sugar-containing carbonated soft drinks (18.0%), and diet sports drinks (20.5%) available for sale.

**Table 1:** School food environment characteristics of participating COMPASS secondary schools (*n* = 78) at Wave 1 (2013/14) within three policy groups: Alberta (*n* = 9), Ontario – Public (*n* = 64), and Ontario – Private (*n* = 5)

Characteristic	Total n (%)	Alberta n (%)	Ontario – Public n (%)	Ontario – Private n (%)	*p* value^a^
*Food outlets present within the school*					
Cafeteria					0.003
Not present	5 (6.4)	2 (22.2)	1 (1.6)	2 (40.0)	
≥ 1 present	73 (93.6)	7 (77.8)	63 (98.4)	3 (60.0)	
Tuck shop					0.036
Not present	69 (88.5)	7 (77.8)	59 (92.2)	3 (60.0)	
≥ 1 present	9 (11.5)	2 (22.2)	5 (7.8)	2 (40.0)	
Beverage vending machines					0.682
No machines present	3 (3.8)	0 (0.0)	3 (4.7)	0 (0.0)	
1 machine present	8 (10.3)	1 (11.1)	6 (9.4)	1 (20.0)	
2 machines present	24 (30.8)	3 (33.3)	21 (32.8)	0 (0.0)	
≥ 3 machines present	43 (55.1)	5 (55.6)	34 (53.1)	4 (80.0)	
*Food outlets present within 1-km buffer of schools*					
Restaurants					0.398
Not present	10 (12.8)	0 (0.0)	9 (14.1)	1 (20.0)	
≥ 1 present	68 (87.2)	9 (100.0)	55 (85.9)	4 (80.0)	
Variety stores					0.086
Not present	45 (57.7)	8 (88.9)	35 (54.7)	2 (40.0)	
1 present	33 (42.3)	1 (11.1)	39 (45.3)	3 (60.0)	
Food stores					0.204
Not present	15 (19.2)	0 (0.0)	15 (23.4)	0 (0.0)	
≥ 1 present	63 (80.8)	9 (100.0)	49 (76.6)	9 (100.0)	
*Contents of beverage vending machines* ^*b*^					
*Sugar-sweetened beverages*					
Sugar-containing carbonated soft drinks (e.g., non-diet Coca-Cola, non-diet Sprite, etc.)					< 0.001
0 types drinks available	64 (82.0)	5 (55.6)	59 (92.2)	0 (0.0)	
1 types drink available	5 (6.4)	0 (0.0)	5 (7.8)	0 (0.0)	
2 types drinks available	3 (3.9)	1 (11.1)	0 (0.0)	2 (40.0)	
≥ 3 types drinks available	6 (7.7)	3 (33.3)	0 (0.0)	3 (60.0)	
Sugar-containing non-carbonated soft drinks (e.g., non-diet lemonade, fruit drinks, iced tea, etc.)					< 0.001
0 types drinks available	36 (46.1)	0 (0.0)	36 (56.2)	0 (0.0)	
1 types drink available	3 (3.9)	1 (11.1)	1 (1.6)	1 (20.0)	
2 types drinks available	11 (14.1)	1 (11.1)	9 (14.1)	1 (20.0)	
≥ 3 types drinks available	28 (35.9)	7 (77.8)	18 (28.1)	3 (60.0)	
Sugar-containing sports drinks (e.g., Gatorade, PowerAde, etc.)					< 0.001
0 types drinks available	67 (85.9)	3 (33.3)	63 (98.4)	1 (20.0)	
1 types drink available	3 (3.8)	2 (22.2)	0 (0.0)	1 (20.0)	
2 types drinks available	2 (2.6)	1 (11.2)	0 (0.0)	1 (20.0)	
≥ 3 types drinks available	6 (7.7)	3 (33.3)	1 (1.6)	2 (40.0)	
Flavoured milk (e.g., strawberry, chocolate milk)					0.625
0 types drinks available	42 (53.8)	6 (66.7)	33 (51.6)	3 (60.0)	
1 types drink available	9 (11.5)	1 (11.1)	8 (12.5)	0 (0.0)	
2 types drinks available	3 (3.9)	1 (11.1)	2 (3.1)	0 (0.0)	
≥ 3 types drinks available	24 (30.8)	1 (11.1)	21 (32.8)	2 (40.0)	
*Non-sugar-sweetened beverages*					
Diet carbonated soft drinks (e.g., Diet Coke, Coke Zero, Sprite Zero, etc.)					0.244
0 types drinks available	22 (28.2)	3 (33.4)	19 (29.7)	0 (0.0)	
1 types drink available	8 (10.3)	1 (11.1)	5 (7.8)	2 (40.0)	
2 types drinks available	8 (10.3)	1 (11.1)	6 (9.4)	1 (20.0)	
≥ 3 types drinks available	40 (51.2)	4 (44.4)	34 (53.1)	2 (40.0)	
Diet non-carbonated soft drinks (e.g., diet lemonade, Fresca, diet iced tea, etc.)					0.285
0 types drinks available	40 (51.3)	6 (66.7)	31 (48.5)	3 (60.0)	
1 types drink available	11 (14.1)	2 (22.2)	7 (10.9)	2 (40.0)	
2 types drinks available	10 (12.8)	0 (0.0)	10 (15.6)	0 (0.0)	
≥ 3 types drinks available	17 (21.8)	1 (11.1)	16 (25.0)	0 (0.0)	
Diet sports drinks (e.g., G2, Powerade Zero, etc.)					0.126
0 types drinks available	62 (79.5)	6 (66.7)	53 (82.8)	3 (60.0)	
1 types drink available	4 (5.1)	1 (11.1)	2 (3.1)	1 (20.0)	
2 types drinks available	4 (5.1)	0 (0.0)	3 (4.7)	1 (20.0)	
≥ 3 types drinks available	8 (10.3)	2 (22.2)	6 (9.4)	0 (0.0)	
Plain white milk					0.851
0 types drinks available	58 (74.4)	8 (88.9)	46 (71.9)	4 (80.0)	
1 types drink available	4 (5.1)	0 (0.0)	4 (6.2)	0 (0.0)	
2 types drinks available	10 (12.8)	1 (11.1)	9 (14.1)	0 (0.0)	
≥ 3 types drinks available	6 (7.7)	0 (0.0)	5 (7.8)	1 (20.0)	
100% fruit juice					0.033
0 types drinks available	11 (14.1)	1 (11.2)	9 (14.1)	1 (20.0)	
1 types drink available	2 (2.6)	0 (0.0)	0 (0.0)	2 (40.0)	
2 types drinks available	32 (41.0)	4 (44.4)	27 (42.2)	1 (20.0)	
≥ 3 types drinks available	33 (42.3)	4 (44.4)	28 (43.7)	1 (20.0)	
Water					0.011
0 types drinks available	21 (26.9)	0 (0.0)	21 (32.8)	0 (0.0)	
1 types drink available	19 (24.4)	4 (44.4)	12 (18.7)	3 (60.0)	
2 types drinks available	23 (29.5)	5 (55.6)	17 (26.6)	1 (20.0)	
≥ 3 types drinks available	15 (19.2)	0 (0.0)	14 (21.9)	1 (20.0)	

^a^Fisher’s Exact Test used to examine differences by policy group

^b^Schools without beverage vending machines (n = 4) were coded as having ‘0 drinks available’ within each beverage category

Note: percentages rounded to sum to 100%

Schools within the three policy groups differed significantly at Wave 1 with respect to their vending machine availability of water, 100% fruit juice, and three SSBs types: sugar-containing soft drinks (carbonated and non-carbonated) and sports drinks (Table 1). Specifically, with the exception of flavoured milk availability (which was similar across the three policy groups), SSB availability was considerably lower in Ontario public schools. For example, nearly all (98.4%) of Ontario public schools had zero sugar-containing sports drinks available in their vending machines, compared to 33.3 and 20.0% of Alberta schools and Ontario private schools, respectively.

### Changes in beverages available for sale within school vending machines

Additional file 1 shows changes in the availability of various beverage categories within schools’ vending machines in the different policy groups. Within Ontario public schools, vending machine beverage availability was generally stable over time. For example, the proportion of Ontario public schools without any sugar-containing carbonated soft drinks, diet carbonated soft drinks, diet sports drinks, and plain white milk fluctuated by less than 5% over time. There was a steady increase in the proportion of Ontario public schools offering ≥1 flavoured milk, from 48.4% in Wave 1 to 59.4% in Wave 3. Conversely, the proportion of schools with bottled water available consistently decreased, from 67.2% in Wave 1 to 59.4% in Wave 3. The changes in beverage availability in Alberta and Ontario private school vending machines were more pronounced. For example, in Alberta the proportion of schools offering diet non-carbonated soft drinks (e.g., diet iced tea, diet lemonade, etc.) increased markedly, from 33.3% in Wave 1 to 88.9% in Wave 3. The availability of sugar-containing carbonated soft drinks fluctuated considerably in Ontario private schools, from 100% of schools having ≥1 drink in Wave 1 to 40% in Wave 2 and 60% in Wave 3.

Ontario public schools generally had a higher proportion of diet drinks available within vending machines compared to their sugar-containing beverage equivalent, while the other policy groups exhibited the opposite trend. For example, at Wave 1, 17.2 and 1.6% of Ontario public schools offered diet sports drinks and sugar-containing sports drinks, respectively, whereas 33.3 and 66.7% of Alberta schools offered diet sports drinks and sugar-containing sports drinks, respectively. With the exception of flavoured milks, SSB availability was lowest in vending machines in Ontario public schools across all waves and generally highest among Ontario private schools.

### Participants’ socio-demographic characteristics and SSB consumption at baseline

Table 2 shows student participants’ socio-demographic characteristics and SSB intake measures at Wave 1. Since the sample included individuals that attended secondary school across Waves 1–3, nearly all participants (97.4%) were in Grades 9 or 10 at Wave 1. Participants reported consuming soft drinks most often (mean 1.73 weekdays) and energy drinks least often (mean 0.15 weekdays). There were significant differences in participants’ SSB intake measures across policy groups. Albertan participants reported a greater rate of SSB consumption across all four outcome measures compared to those in Ontario.

**Table 2:** Socio-demographic and behavioural characteristics of participating COMPASS secondary school students (*n* = 7679) from Alberta (*n* = 497), Ontario – Public (*n* = 6674), and Ontario – Private (*n* = 508) schools at Wave 1 (2013/14)

Characteristic	Total n (%)	Alberta n (%)	Ontario – Public n (%)	Ontario – Private n (%)	*p* value^a^
*Socio-demographic and behavioural*					
Gender					< 0.001
Female	4110 (53.5)	292 (58.8)	3642 (54.6)	176 (34.7)	
Male	3569 (46.5)	205 (41.2)	3032 (45.4)	332 (65.3)	
Grade					< 0.001
9	4138 (53.8)	158 (31.8)	3717 (55.7)	263 (51.8)	
10	3346 (43.6)	334 (67.2)	2773 (41.6)	239 (47.0)	
11	191 (2.5)	5 (1.0)	181 (2.7)	5 (1.0)	
12	4 (0.1)	0 (0.0)	3 (0.0)	1 (0.2)	
Ethnicity					< 0.001
White	5983 (77.9)	385 (77.5)	5245 (78.6)	353 (69.5)	
Aboriginal	143 (1.9)	42 (8.4)	101 (1.5)	22 (4.3)	
Asian	429 (5.6)	18 (3.6)	358 (5.4)	53 (10.4)	
Black	226 (2.9)	4 (0.8)	200 (3.0)	0 (0.0)	
Latin	109 (1.4)	2 (0.4)	99 (1.5)	8 (1.6)	
Other	789 (10.3)	46 (9.3)	671 (10.0)	72 (14.2)	
Weekly spending money					< 0.001
$0	1625 (21.2)	85 (17.1)	1436 (21.5)	104 (20.5)	
$1–$20	2978 (38.8)	129 (26.0)	2659 (39.9)	190 (37.4)	
$21–$100	1607 (20.9)	126 (25.3)	1360 (20.4)	121 (23.8)	
> $100	412 (5.4)	54 (10.9)	342 (5.1)	16 (3.1)	
I don’t know/missing	1057 (13.7)	103 (20.7)	877 (13.1)	77 (15.2)	
Weight status					< 0.001
Underweight	107 (1.4)	6 (1.2)	91 (1.4)	10 (2.0)	
Healthy weight	4437 (57.8)	268 (53.9)	3826 (57.3)	343 (67.5)	
Overweight	1045 (13.6)	73 (14.7)	900 (13.5)	72 (14.2)	
Obese	429 (5.6)	35 (7.1)	382 (5.7)	12 (2.3)	
Missing	1661 (21.6)	115 (23.1)	1475 (22.1)	71 (14.0)	
Truancy					< 0.001
Skipped 0 classes in last four weeks	6730 (87.6)	400 (80.5)	5864 (87.9)	466 (91.7)	
Skipped ≥1 classes in last four weeks	949 (12.4)	97 (19.5)	810 (12.1)	42 (8.3)	
Weight goal					< 0.001
Not trying to do anything about weight	1851 (24.1)	129 (26.0)	1599 (24.0)	123 (24.2)	
Gain weight	1094 (14.3)	55 (11.0)	930 (13.9)	109 (21.5)	
Lose weight	3136 (40.8)	224 (45.1)	2732 (40.9)	180 (35.4)	
Stay the same weight	1598 (20.8)	89 (17.9)	1413 (21.2)	96 (18.9)	
*Weekday SSB consumption* ^*b*^	Mean ± SD	Mean ± SD	Mean ± SD	Mean ± SD	p value ^c^
Freq. of consuming soft drinks	1.73 ± 1.70	1.92 ± 1.74	1.73 ± 1.70	1.64 ± 1.58	0.029
Freq. of consuming sweetened coffees/teas	1.15 ± 1.58	1.30 ± 1.63	1.14 ± 1.57	1.11 ± 1.60	0.021
Freq. of consuming energy drinks	0.15 ± 0.60	0.34 ± 0.92	0.14 ± 0.59	0.07 ± 0.38	< 0.001
Composite SSB score^d^	3.03 ± 2.65	3.57 ± 2.92	3.00 ± 2.64	2.83 ± 2.43	< 0.001

SSB, sugar-sweetened beverage

^a^Chi square analyses used to examine differences by policy group

^b^Number of days in a typical school week (Mon.-Fri., 0–5 days)

^c^Kruskal-Wallis test used to examine differences by policy group

^d^A composite score, ranging from 0 to 15, representing the sum of participants’ weekday rates of consuming the three distinct SSB categories

Note: percentages rounded to sum to 100%

### Changes in participants’ rate of SSB consumption

**Fig. 1:**
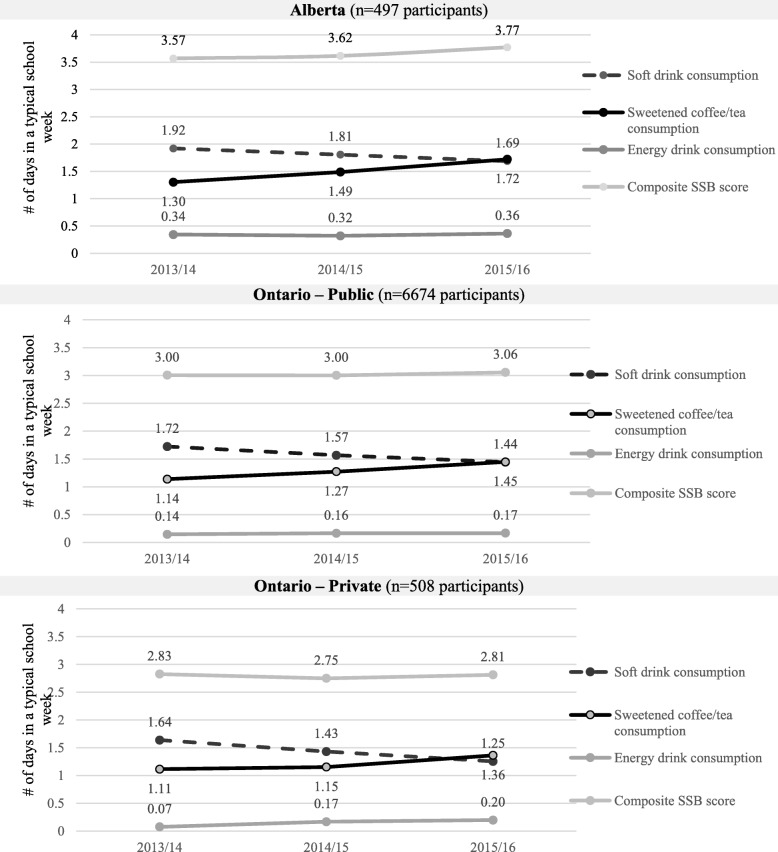
Changes between Waves 1–3 (2013/14–2015/16) in sugar-sweetened beverage consumption-related measures (Plotted values represent mean rate of the SSB consumption measure among participants in each policy group at each wave of data) among COMPASS participants attending schools within three policy groups: Alberta (*n* = 9 schools), Ontario – Public (*n* = 64 schools), and Ontario – Private (*n* = 5 schools)

The direction of change in participants’ weekday rate of SSB consumption across Waves 1–3 was comparable across policy groups (Fig. 1); participants’ rate of soft drink consumption decreased while their rate of sweetened coffee/tea consumption increased. Participants’ rate of energy drink consumption and composite SSB score remained fairly steady, showing relatively smaller increases over time. Across all four outcome measures and time points, participants in Alberta reported a higher rate of SSB intake than their Ontario counterparts. Ontario public school students reported more frequent SSB consumption at all time points compared to private school students, with the exception of energy drink intake, which was higher among private school students in Waves 2–3.

### Modeling predictors of beverage availability

Table 3 displays the associations between school-level characteristics and vending machine availability of each of the ten beverage categories. The effect of policy group was significantly (*P <* .05) associated with the availability of sugar-containing soft drinks (carbonated and non-carbonated) and sports drinks; these SSBs were considerably more available in Alberta and Ontario private schools, relative to Ontario public schools, after adjusting for control variables. The models demonstrated that beverage availability generally did not vary significantly over time; however, with each wave of data, the odds of sugar-containing non-carbonated soft drinks being available in schools’ vending machines decreased significantly (*P <* .01).

**Table 3:** Odds ratios for school characteristics associated with availability vs. non-availability of several beverage categories in school vending machines in secondary schools (*n* = 78) representing three policy groups in Alberta and Ontario that participated in Waves 1–3 (2013/14–2015/16) of the COMPASS study

Characteristic	Beverage categories Adjusted odds ratio^a^ (95% CI)									
	Sugar-containing carbonated soft drinks	Sugar-containing non-carbonated soft drinks	Sugar-containing sports drinks	Flavoured milk	Diet carbonated soft drinks	Diet non-carbonated soft drinks	Diet sports drinks	Plain white milk	100% fruit juice	Water
Policy group										
Ontario – Public	1.0	1.0	1.0	1.0	1.0	1.0	1.0	1.0	1.0	1.0
Ontario –Private	28.03 (5.44–144.39)***	26.00 (2.82–239.77)**	111.14 (10.69–1155.51)***	0.50 (0.08–3.34)	0.45 (0.10–2.07)	0.61 (0.07–5.35)	13.75 (1.97–96.20)**	0.81 (0.09–7.03)	0.19 (0.01–2.51)	1.00 (0.11–8.85)
Alberta	16.81 (2.54–111.11)**	6.25 (1.12–34.90)*	10.18 (2.39–43.38)**	0.43 (0.11–1.67)	1.02 (0.27–3.81)	1.10 (0.32–3.74)	0.98 (0.16–5.89)	0.20 (0.02–2.08)	2.09 (0.48–9.05)	2.90 (0.48–17.34)
Wave^b^	0.86 (0.54–1.36)	0.77 (0.64–0.92)**	1.37 (0.82–2.29)	1.17 (0.96–1.42)	0.88 (0.71–1.11)	1.14 (0.91–1.43)	0.95 (0.77–1.18)	0.98 (0.73–1.31)	0.92 (0.62–1.35)	0.81 (0.69–0.95)

^a^Odds adjusted for all other variables in the column, in addition to geographic location, school neighbourhood median income, and the presence of restaurants, variety stores, and food stores within schools’ 1-km circular buffer

^b^Wave was treated as a continuous variable, where Wave 1 = 0, Wave 2 = 1, and Wave 3 = 2

*P < 0.05, **P < 0.01, ***P < 0.001

### Modeling predictors of SSB intake

Few of the ten measures of SSB availability were significantly associated (*P <* .20) with the four SSB consumption-related outcomes in the univariate analyses, and thus few were retained for inclusion in the joint multivariate models (Table 4).

**Table 4:** Univariate analyses for vending machine beverage availability variables in relation to students’ weekday consumption of SSBs among secondary school students (*n* = 7679) in Alberta and Ontario participating in Waves 1–3 (2013/14–2015/16) of the COMPASS study

Variable	Weekday SSB consumption^a^ Rate^b^ (95% CI)			
	Composite SSB score^c^	Soft drink	Sweetened coffees/teas	Energy drinks
*SSBs*				
Sugar-containing carbonated soft drinks (e.g., non-diet Coca-Cola, non-diet Sprite, etc.)				
0 drinks available	REF	REF	REF	REF
≥1 drinks available	1.01 (0.97–1.05)	1.00 (0.94–1.06)	1.03 (0.95–1.11)	0.99 (0.81–1.21)
Sugar-containing non-carbonated soft drinks (e.g., non-diet lemonade, fruit drinks, iced tea, etc.)				
0 drinks available	REF	REF	REF	REF
≥1 drinks available	1.01 (0.98–1.04)	1.05 (1.01–1.10)*	0.97 (0.91–1.03)	1.04 (0.89–1.21)
Sugar-containing sports drinks (e.g., Gatorade, PowerAde, etc.)				
0 drinks available	REF	REF	REF	REF
≥1 drinks available	**1.03 (0.99–1.07)**	0.98 (0.91–1.06)	**1.06 (0.97–1.15)**	1.21 (0.89–1.63)
Flavoured milk (e.g., strawberry, chocolate milk)				
0 drinks available	REF	REF	REF	REF
≥1 drinks available	**0.97 (0.94–1.01)**	0.94 (0.89–0.99)*	1.03 (0.97–1.09)	**0.96 (0.73–1.00)**
*Non-SSBs*				
Diet carbonated soft drinks (e.g., Diet Coke, Coke Zero, Sprite Zero, etc.)				
0 drinks available	REF	REF	REF	REF
≥1 drinks available	0.98 (0.95–1.02)	0.99 (0.93–1.06)	0.98 (0.91–1.05)	0.91 (0.76–1.08)
Diet non-carbonated soft drinks (e.g., diet lemonade, Vitaminwater Zero, diet iced tea, etc.)				
0 drinks available	REF	REF	REF	REF
≥1 drinks available	1.00 (0.98–1.03)	0.99 (0.94–1.04)	1.01 (0.95–1.07)	**1.10 (0.96–1.25)**
Diet sports drinks (e.g., G2, Powerade Zero, etc.)				
0 drinks available	REF	REF	REF	REF
≥1 drinks available	**1.03 (0.99–1.09)**	1.02 (0.94–1.11)	1.03 (0.95–1.11)	1.10 (0.85–1.43)
Plain white milk				
0 drinks available	REF	REF	REF	REF
≥1 drinks available	**0.97 (0.94–1.01)**	0.99 (0.93–1.06)	**0.95 (0.90–1.01)**	**0.89 (0.75–1.05)**
100% fruit juice				
0 drinks available	REF	REF	REF	REF
≥1 drinks available	1.02 (0.97–1.07)	1.02 (0.94–1.10)	1.02 (0.93–1.11)	1.01 (0.84–1.21)
Water				
0 drinks available	REF	REF	REF	REF
≥1 drinks available	**1.03 (0.99–1.07)**	1.07 (1.01–1.13)*	1.00 (0.93–1.07)	0.98 (0.83–1.16)

SSB, sugar-sweetened beverage

^a^Number of weekdays participants reported consuming SSBs in a typical school week (Mon.-Fri., 0–5 days)

^b^Rates represents the exponentiated beta coefficients; bold values are statistically significant (*P* < 0.20)

^c^A composite score, ranging from 0 to 15, representing the sum of participants’ weekday rates of consuming the three distinct SSB categories

*P < 0.05, **P < 0.01, ***P < 0.001

When comparing the three series of multivariate models developed (i.e., vending machine beverage availability block only, policy group block only, and both blocks together), it was clear that the interpretation and significance of variables of interest did not differ across the series of models for each outcome. Table 5 displays the four multivariate models that contained both the vending machine beverage availability and policy group blocks. Controlling for all other variables, there were significant (*P < .*05) differences in participants’ SSB consumption outcomes between Ontario public schools (the reference group) and the other policy groups in three of the four models. Specifically, relative to the Ontario public school students, attending school in Alberta was associated with a higher composite SSB score and rate of energy drink consumption, whereas attending an Ontario private school was associated with less frequent soft drink intake. The effect of wave was significant across models and suggested that, after controlling for all other variables, participants’ rate of SSB consumption decreases as they progress through secondary school, with the exception of sweetened coffees/teas, which participants consumed more often with time. Few of the beverage availability variables retained their significance in the models. The availability of bottled water was significantly (*P < .*001) associated with a higher rate of soft drink consumption and composite SSB score.

**Table 5:** Final multivariate models describing correlates of weekday consumption of three varieties of SSBs among secondary school students (*n* = 7679) in Alberta and Ontario participating in Waves 1–3 (2013/14–2015/16) of the COMPASS study

Variable	Weekday SSB consumption^a^ Rate^b^ (95% CI)			
	Composite SSB score^c^	Soft drink	Sweetened coffees/teas	Energy drinks
*Policy group*				
Ontario – Public	REF	REF	REF	REF
Ontario – Private	0.96 (0.90–1.03)	0.92 (0.86–0.99)*	0.97 (0.88–1.06)	1.01 (0.63–1.61)
Alberta	1.11 (1.00–1.24)*	1.09 (0.98–1.20)	1.06 (0.91–1.23)	1.40 (1.08–1.83)*
Wave	0.96 (0.95–0.98)***	0.89 (0.88–0.91)***	1.06 (1.04–1.09)***	0.90 (0.84–0.97)**
*SSBs*				
Sugar-containing carbonated soft drinks (e.g., non-diet Coca-Cola, non-diet Sprite, etc.)				
0 drinks available	–	–	–	–
≥1 drinks available	–	–	–	–
Sugar-containing non-carbonated soft drinks (e.g., non-diet lemonade, fruit drinks, iced tea, etc.)				
0 drinks available	–	REF	–	–
≥1 drinks available	–	0.97 (0.93–1.01)	–	–
Sugar-containing sports drinks (e.g., Gatorade, PowerAde, etc.)				
0 drinks available	REF	–	REF	–
≥1 drinks available	0.98 (0.92–1.04)	–	1.02 (0.94–1.12)	–
Flavoured milk (e.g., strawberry, chocolate milk)				
0 drinks available	REF	REF	–	REF
≥1 drinks available	1.00 (0.96–1.04)	0.98 (0.94–1.03)	–	0.94 (0.78–1.14)
*Non-SSBs*				
Diet carbonated soft drinks (e.g., Diet Coke, Coke Zero, Sprite Zero, etc.)				
0 drinks available	–	–	–	–
≥1 drinks available	–	–	–	–
Diet non-carbonated soft drinks (e.g., diet lemonade, Vitaminwater Zero, diet iced tea, etc.)				
0 drinks available	–	–	–	REF
≥1 drinks available	–	–	–	0.97 (0.84–1.12)
Diet sports drinks (e.g., G2, Powerade Zero, etc.)				
0 drinks available	REF	–	–	–
≥1 drinks available	0.98 (0.93–1.04)	–	–	–
Plain white milk				
0 drinks available	REF	–	REF	REF
≥1 drinks available	0.99 (0.95–1.03)	–	1.00 (0.95–1.05)	1.01 (0.82–1.24)
100% fruit juice				
0 drinks available	–	–	–	–
≥1 drinks available	–	–	–	–
Water				
0 drinks available	REF	REF	–	–
≥1 drinks available	1.07 (1.03–1.12)***	1.08 (1.03–1.13)**	–	–

SSB, sugar-sweetened beverage

^a^Number of weekdays participants reported consuming SSBs in a typical school week (Mon.-Fri., 0–5 days)

^b^ Rates represents the exponentiated beta coefficients; rates adjusted for all other variables in the column, in addition to student- and school-level control variables

^c^A composite score, ranging from 0 to 15, representing the sum of participants’ weekday rates of consuming the three distinct SSB categories

--- excluded from model during univariate analyses screening

*P < 0.05, **P < 0.01, ***P < 0.001

## Discussion

This study examined temporal changes in the contents of the beverage vending machines within secondary schools in Alberta and Ontario and students’ rate of days of SSB consumption, in order to assess the implementation and potential impact of distinct school nutrition policies in these two Canadian provinces.

### Beverage availability in secondary schools

Most SSB categories we examined were less available in school vending machines in Ontario public schools compared to Alberta schools, which may reflect the mandatory versus voluntary nature of the provincial school nutrition policies in these jurisdictions [30]. The exception to this was flavoured milks, which were more available in Ontario public schools than the other policy groups. This finding likely reflects flavoured milks being generally permitted for sale within Ontario public secondary schools, since flavoured low-fat milk and milk-based beverages are considered ‘sell-most’ items within *P/PM 150*, provided they contain ≤28 g sugar/serving [25].. Although flavoured milks (e.g., chocolate milk) often contain a high quantity of added sugar, some differentiate these beverages from other SSBs, since they are also a source of calcium, protein, and vitamin D. A natural experiment in the United States identified that policies restricting the in-school sale of SSBs were associated with lower in-school SSB access, but only if all SSBs were banned from school food outlets (i.e., not just sodas) [50]. Since policy comprehensiveness was not assessed in the current study, we cannot assert that this drove differences in beverage availability across policy groups. Our observation that SSBs were often markedly less available in vending machines in Albertan schools compared to Ontario private schools provides evidence that even voluntary provincial school nutrition policies may be more effective at restricting the in-school sale of SSBs than the absence of these polices. Overall, our findings are consistent with previous Canadian research that suggests that provincial school nutrition policies can effectively limit in-school SSB availability [51], particularly when they are mandatory [30].

Other notable policy group differences included a greater availability of diet beverages and lower availability of bottled water in Ontario public schools. Diet beverages are permitted under *P/PM 150* guidelines; however, there is no consensus on the health impacts and acceptability of non-nutritive sweeteners, both within Canadian school food guidelines [23] and recent research syntheses [52, 53]. The decreased availability of bottled water in Ontario vending machines may reflect environmental movements to limit use of plastic bottled water in favour of water fountains in schools (e.g., “Ban the Bottle” campaigns underway in schools globally). Previous research has identified unintended consequences of these initiatives in some settings, including significant increases in the number of plastic bottles being shipped to schools (thereby entering the waste stream) and greater consumption of less healthy bottled beverages among students [54]. This does not, however, explain our finding that the availability of bottled water was associated with a higher composite SSB score and frequency of soft drink intake among participants, which was contrary to expectations. We are unaware of previous research that has identified similar associations, although it is unlikely that the association is causal.

Although Ontario public schools had a significantly lower availability of most SSBs in their vending machines compared to the other policy groups, the presence of these products reflects non-compliance with *P/PM 150*, as noted in previous studies [29], including other analyses of COMPASS data [26, 30]. Reasons for non-compliance with school food policies include school stakeholders’ perceptions of lower revenue generation, time and resource constraints, higher priced policy compliant foods [49], as well as ambiguities within policy recommendations, a lack of support with implementation, and limited policy monitoring [23, 26, 55]. Greater enforcement and additional supports to help school stakeholders to understand and implement the policies as intended would likely increase policy compliance. Our finding that 90 + % of Ontario public schools had no sugar-containing carbonated soft drinks and sports drinks available in their vending machines over three school years is a positive result, and demonstrates schools’ adherence to *P/PM 150*. There is further encouragement in our finding that SSB availability did not shift significantly over the study period, which disproved our hypothesis that policy adherence would decrease with time. This stability in policy adherence with time has been noted in a previous evaluation of *P/PM 150* [29], though is inconsistent with other research that identifies decreased school nutrition policy adherence in the years post- implementation [26]. These differences likely reflect differing methodologies, including how policy adherence was measured. It is important to note the absence of pre-policy data precludes the ability to conclude that the relatively low proportion of Ontario public schools with these SSBs available and stability in vending machine beverage offerings reflects *P/PM 150* implementation, as these findings may be an artefact of pre-policy conditions.

### Changes in adolescents’ SSB consumption over time

Across all three policy groups, participants’ overall frequency of SSB consumption remained fairly stable as they progressed through secondary school, although their intake of certain SSB categories shifted. Notably, sweetened coffees/teas displaced soft drinks as the most frequently consumed SSB among participants. Many varieties of sweetened coffees/teas (e.g., speciality coffees drinks) contain as much or more sugar than sodas [56] and considerably more caffeine [57]. The high added sugar content of these and other SSBs is worrying due to associations between fructose (i.e., the central component of added sugars) and numerous chronic conditions, including hypertension, insulin resistance, diabetes, and liver damage [58]. The concerns related to frequent sweetened coffee/tea consumption among adolescents are further compounded by the beverages’ high caffeine content particularly since recent estimates of adolescents’ caffeine consumption exceed Health Canada’s recommendations for this age group [57]. Adolescents’ caffeinated beverage consumption has increased significantly over recent decades [59], mirroring increases in the per capital sales volume for sweetened coffee/teas in Canada [1]. A recent Canadian study identified various reasons for adolescents’ consumption of caffeinated beverages, including parental and peer role modeling, a desire to feel/appear more mature, energy provision, and to remain alert for academic or social activities [60]. Future research should continue to monitor trends in adolescents’ SSB intake, and examine the specific products youth consume, the sources of these beverages, and their associated health effects.

### Provincial school nutrition policy impact

This study did not detect a significant association between beverage availability in school vending machines and adolescents’ frequency of SSB intake. The lack of identified association may reflect methodological limitations of this study (e.g., the examination of only one type of school food outlet, conservative measures of SSB intake based on frequency of days as opposed to volume or number of servings, lack of measure of source of beverages consumed, etc.), as opposed to ineffectiveness of provincial school nutrition policies. Indeed, findings from a recent systematic review [22] and previous Canadian evaluation studies [61, 62] identify that school nutrition policies can have a favourable impact on students’ dietary behaviours. However, our results are consistent with previous research in Canada and elsewhere that report that features of the school food environment have a limited impact on students’ dietary outcomes [19, 31–33, 63].

There are several reasons why, even with perfect school-level compliance, school nutrition policies may be limited in their ability to moderate adolescents’ SSB intake. Vine et al. found that following *P/PM 150*, schools noted an increase in students leaving school to purchase meals/snacks at neighbouring food outlets [49, 64]. A recent COMPASS study identified that students reported purchasing snacks from school vending machines an average of 0.3 days in a typical school week, which was considerably less often than the number of days they made purchases from fast-food places/restaurants, and convenience food outlets off-school property [20]. Indeed, when students are restricted in the foods/beverages they can access during school, they may compensate by consuming more of these items in other settings [61, 65, 66]. Further, previous research identifies that SSB intake is primarily driven by socio-cultural and intrapersonal-level (vs. school-level) factors [19, 33], which is reflected in the very limited (≤2%) proportion of the variability in students’ rate of SSB intake accounted for by school-level differences [20]. This earlier research underscores the value in comprehensive school-based nutrition interventions (i.e., interventions that address numerous determinants of dietary behaviours, via nutrition education, initiatives to improve students’ food skills, school nutrition policies, built environment changes, etc.), as well as broader population-level efforts (e.g., taxation of sugary drinks, marketing restrictions, etc.), to communicate a consistent health-reinforcing message through various channels to support behaviour change [61, 66, 67].^.^

### Study strengths and limitations

The strengths of this study include its longitudinal design, large and diverse school and student samples, cross-jurisdictional comparisons, inclusion of private schools (i.e., given the limited research on the school food environment in this school setting) [35], and timely examinations of Canadian adolescents’ SSB intake, which is particularly valuable in light of forthcoming changes to national food guidelines and nutrition policies (e.g., restrictions of food/beverage marketing to children, labelling changes, etc.) in Canada. In particular, Canada’s revised national food guidelines are set for release later in 2018, and their release is likely to motivate subsequent amendments to provincial school nutrition policies, since previous research has identified that many Canadian jurisdictions base their nutrition policy guidelines on the Food Guide recommendations [23]. As such, the present study’s results represent important baseline data, enabling future pre−/post-assessments of the impact these policy changes have on both school food environment characteristics and students’ frequency of SSB intake using the COMPASS research platform.

This study has important limitations, some of which reflect the fact that the COMPASS study was not specifically designed to provide a detailed assessment of the beverages available for sale within schools or students’ SSB consumption patterns. We only assessed beverage availability within school vending machines, which is key limitation, since recent COMPASS data identify that students’ frequency of vending machine use was relatively low compared to frequency of bringing a home-packed lunch or purchasing lunch from the school cafeteria or fast food places/restaurants [20]. However, data on beverages for sale within school cafeterias or tuck shops were not consistently available for various reasons, including cafeterias and tuck shops being closed during data collections and COMPASS researchers often being denied permission to enter and/or photograph school cafeterias, particularly those operated by external private companies. Measures of SSB consumption were not validated and likely underestimate participants’ true SSB intake due to the unit of measure used (i.e., as compared to volume or number of servings of SSBs consumed), the self-reported nature of this and other student-level measures (e.g., height and weight), and since certain SSBs (e.g., flavoured milks) are not captured on the questionnaire. Likewise, there were no measures of source of beverages consumed (i.e., to capture intake of beverages at home vs. at school vs. in other settings), thus caution must be taken when interpreting associations between students’ frequency of SSB intake and school food environment characteristics. Further, there was imperfect alignment between of SSB outcome measures and vending machine drink categories. For example, the “sugar-containing non-carbonated soft drink” category comprised both soft drinks (e.g., < 100% fruit drinks) and sweetened coffees/teas (e.g., iced tea). Likewise, we examined the numbers of each beverage type in vending machine, as opposed to the proportion of total beverage vending machine items each beverage type represents (i.e., which P/PM 150 specifies in its guidelines through “sell most”, “sell less”, and “not permitted for sale” categorizations), making our assessments of beverage machine availability an imperfect representation of compliance with provincial school nutrition policies. This study is also limited by the relatively small number Albertan and Ontario private schools, which reflect the fact that most Canadian schools are publicly-funded and COMPASS’ inception in Ontario. Finally, this study lacks data pre-implementation of provincial school nutrition policies in Alberta and Ontario, precluding the ability to examine changes in beverage availability and/or students’ rate of SSB intake as a direct result of policy implementation.

## Conclusions

The mandatory nutrition policy implemented in Ontario public schools is associated with a substantially lower availability of SSBs in secondary school vending machines, compared to the voluntary policies in Alberta and Ontario private schools. However, the lack of pre-policy data precludes the ability to state that beverage availability reflected policy effectiveness. Although policy compliance in Ontario public schools was imperfect—particularly with respect to sugar-containing non-carbonated soft drinks (i.e., primarily fruit drinks and iced teas) —adherence to the policy was generally high and did not decrease over time. SSB consumption was significantly lower in Ontario public and private schools, although the current study did not detect a direct association between frequency of consumption and SSB availability. Overall, the findings provide support for mandatory versus voluntary school nutrition policies, as well as the need for comprehensive school- and broader population-level efforts that address other factors that influence adolescents’ dietary choices (e.g., individuals’ diet-related values, knowledge, attitudes, and food skills), in addition to the accessibility of unhealthy vs. healthy food and beverages.

## Additional file

Additional file 1: Changes in the number of types of beverages available in vending machines within participating COMPASS secondary schools (*n* = 78) within three policy groups: Alberta (*n* = 9), Ontario – Public (*n* = 64), and Ontario – Private (*n* = 5). (DOCX 53 kb)

## Abbreviations

*ANGCY*: *Alberta Nutrition Guidelines for Children & Youth*
BMI: body mass index
COMPASS: Cannabis use, Obesity Mental health Physical activity Alcohol use Smoking Sedentary behaviour
Co-SEA: COMPASS School Environment Application
DMTI: Desktop Mapping Technologies Inc.
*P/PM 150*: *Policy/program Memorandum no. 150*
SSB: Sugar sweetened beverage
