# Supplementary material for: Examining changes in school vending machine beverage availability and sugar-sweetened beverage intake among Canadian adolescents participating in the COMPASS study: a longitudinal assessment of provincial school nutrition policy compliance and effectiveness
Source: Int J Behav Nutr Phys Act. 2018 Nov 27;15:121. doi: 10.1186/s12966-018-0754-5 (PMC6257956; doi:10.1186/s12966-018-0754-5)
Supplement: Supplementary file 1 — Changes in the number of types of beverages available in vending machines within participating COMPASS secondary schools (n = 78) within three policy groups: Alberta (n = 9), Ontario – Public (n = 64), and Ontario – Private (n = 5). (DOCX 53 kb) [file 12966_2018_754_MOESM1_ESM.docx]

**Additional file 1:** Changes in the number of types of beverages available in vending machines within participating COMPASS secondary schools (n=78) within three policy groups: Alberta (n=9), Ontario – Public (n=64), and Ontario – Private (n=5).

| **SSBs** |
| --- |
| Sugar-containing carbonated soft drinks |
|  |
| Sugar-containing non-carbonated soft drinks |
|  |
| Sugar-containing sports drinks |
|  |
| Flavoured milk |
|  |
| **Non-SSBs** |
| Diet carbonated soft drinks |
|  |
| Diet non-carbonated soft drinks |
|  |
| Diet sports drinks |
|  |
| Plain white milk |
|  |
| 100% fruit juice |
|  |
| Water |
|  |
